# Glycosidic scaffold bearing multiple galloyl moieties from pomegranate disrupts transthyretin amyloids

**DOI:** 10.1016/j.isci.2025.114170

**Published:** 2025-11-21

**Authors:** Asuka Kagami, Nami Hashimoto, Ryoko Sasaki, Yutaro Fukushima, Hari Prasad Devkota, Shoya Tanaka, Mikiyo Wada, Kunitoshi Yamanaka, Shiori Yamakawa, Shogo Misumi, Takeshi Yokoyama, Mineyuki Mizuguchi, Takashi Sato, Teruya Nakamura, Shunsuke Kotani, Mary Ann Suico, Hirofumi Kai, Mitsuharu Ueda, Tsuyoshi Shuto

**Affiliations:** 1 Department of Molecular Medicine, Graduate School of Pharmaceutical Sciences, Kumamoto University, 5-1 Oe-Honmachi, Chuo-ku, Kumamoto 862-0973, Japan; 2 Program for Fostering Innovators to Lead a Better Co-being Society, Kumamoto University, 2-39-1 Kurokami, Chuo-ku, Kumamoto 862-8555, Japan; 3 Health Life Science S-HIGO Professional Fellowship Program, Kumamoto University, 2-39-1 Kurokami, Chuo-ku, Kumamoto 862-8555, Japan; 4 Department of Instrumental Analysis, Graduate School of Pharmaceutical Sciences, Kumamoto University, 5-1 Oe-Honmachi, Chuo-ku, Kumamoto 862-0973, Japan; 5 Faculty of Humanities and Social Sciences, Kumamoto University, 2-40-1 Kurokami, Chuo-ku, Kumamoto 860-8555, Japan; 6 Department of Molecular Cell Biology, Institute of Molecular Embryology and Genetics, Kumamoto University, 2-2-1 Honjo, Chuo-ku, Kumamoto 860-0811, Japan; 7 Department of Neurology, Graduate School of Medical Sciences, Kumamoto University, 1-1-1 Honjo, Chuo-ku, Kumamoto 860-8556, Japan; 8 Department of Environmental and Molecular Health Sciences, Graduate School of Pharmaceutical Sciences, Kumamoto University, 5-1 Oe-Honmachi, Chuo-ku, Kumamoto 862-0973, Japan; 9 Global Center for Natural Resources Sciences, Faculty of Life Sciences, Kumamoto University, 5-1 Oe-Honmachi, Chuo-ku, Kumamoto 862-0973, Japan; 10 Faculty of Pharmaceutical Sciences, University of Toyama, 2630 Sugitani, Toyama 930-0194, Japan; 11 Department of Analytical and Biophysical Chemistry, Graduate School of Pharmaceutical Sciences, Kumamoto University, 5-1 Oe-Honmachi, Chuo-ku, Kumamoto 862-0973, Japan; 12 Department of Structural Biology, Graduate School of Pharmaceutical Sciences, Kumamoto University, 5-1 Oe-Honmachi, Chuo-ku, Kumamoto 862-0973, Japan

**Keywords:** Health sciences, Natural product chemistry, Biological sciences, Natural product biochemistry

## Abstract

Transthyretin (TTR) amyloidosis involves TTR misfolding and aggregation, causing systemic organ dysfunction. Current therapies stabilize TTR tetramers or reduce TTR production but are less effective against existing amyloid deposits. Here, we identified 1,2,3,4,6-penta-*O*-galloyl-β-D-glucose (PGG), derived from pomegranate (*Punica granatum* L.) leaf-and-branch extracts (PGL), as a disruptor of preformed TTR amyloid fibrils. PGG disaggregated fibrils formed by V30M and wild-type (WT) TTR, which are linked to hereditary Transthyretin Amyloidosis (ATTR) (ATTRv) and ATTRwt, respectively. Structure-activity relationship studies showed galloyl moieties are essential. In *Caenorhabditis elegans*-expressing human TTR_81-127_, PGG reduced TTR aggregation and extended both lifespan and healthspan. PGG selectively targeted TTR amyloids without affecting amyloid-β fibrils, indicating specificity. PGG disrupted TTR amyloid fibrils isolated from patient cardiac tissue. These findings suggest that PGG shows therapeutic potential for TTR amyloidosis by directly targeting and disrupting pathogenic amyloid aggregates.

## Highlights

• 1,2,3,4,6-penta-*O*-galloyl-β-glucose (PGG) disrupts TTR but not Aβ amyloid fibrils
• Galloyl groups are essential for PGG’s amyloid-disrupting activity
• PGG reduces TTR aggregates and extends lifespan in a *C. elegans* TTR model
• PGG disaggregates amyloid fibrils derived from ATTRv patient tissues

## Graphical abstract

**Figure:**
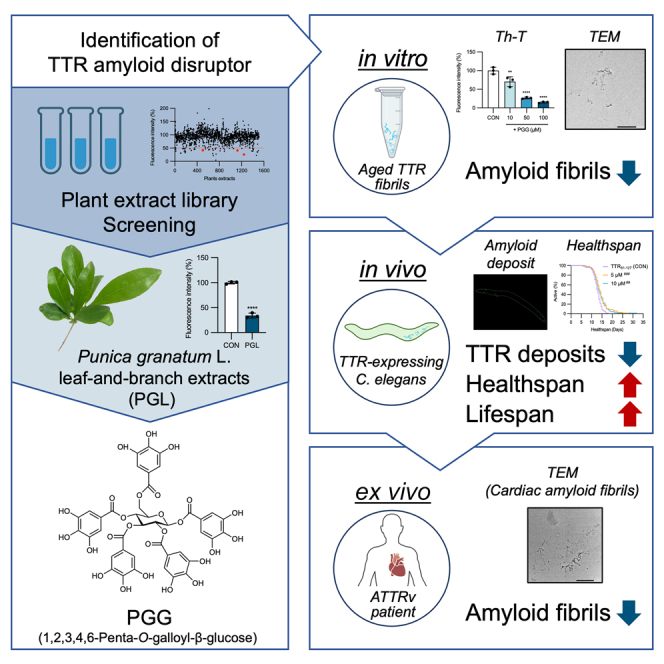

## Introduction

Humans have long utilized compounds from plants, microorganisms, marine organisms, insects, and minerals to address unmet medical challenges.^1^^,^^2^^,^^3^ Consequently, a substantial proportion of modern pharmaceuticals originates from natural products, and surveys indicate that approximately 80% of the global population uses some form of traditional, complementary, and integrative medicine, usually alongside biomedicine.^4^ Natural products are particularly appealing for drug discovery due to their diverse chemical scaffolds, varied substituent patterns, and distinctive functional groups—features often surpassing synthetic analogues in complexity and biological activity.^5^^,^^6^^,^^7^ Numerous undiscovered therapeutic candidates likely remain hidden within natural resources.

ATTR amyloidosis is a debilitating condition characterized by amyloid fibril formation arising from misfolded transthyretin (TTR), resulting in progressive organ dysfunction.^8^^,^^9^ TTR, predominantly synthesized in the liver, typically circulates as a stable tetramer transporting thyroxine and retinol-binding protein. However, the dissociation of TTR tetramers into unstable monomers leads to amyloidogenic aggregation and insoluble fibril deposition, causing organ impairment characteristic of ATTR amyloidosis.^9^^,^^10^ Genetic mutations and age-related destabilization promote tetramer dissociation, yielding hereditary (ATTRv) and wild-type (WT) (ATTRwt) disease, respectively.^11^

ATTRv amyloidosis involves more than 120 identified TTR mutations; the Val30Met mutation is the most prevalent globally, with notable endemic foci in Portugal, Brazil, Sweden, and Japan.^11^^,^^12^ Amyloid fibrils primarily accumulate in peripheral nerves, manifesting clinically as numbness, lower extremity pain, and autonomic dysfunction (e.g., chronic constipation, diarrhea, and orthostatic hypotension), typically presenting between ages 20 and 30. In contrast, ATTRwt amyloidosis arises from age-associated instability of WT TTR without genetic mutations. Approximately 25% of individuals aged over 80 exhibit cardiac amyloid deposition, leading to heart failure and arrhythmias.^13^ Early detection remains challenging because definitive diagnosis often requires invasive cardiac biopsies and amyloid-specific staining, contributing to diagnostic delay and underdiagnosis.^14^

Current treatment approaches include liver transplantation,^15^ TTR-directed small interfering RNA therapeutics such as patisiran that reduce TTR production,^16^ and tetramer stabilizers like Tafamidis that prevent tetramer dissociation.^17^ In addition, preclinical studies have reported that natural product-derived polyphenols inhibit TTR amyloid fibril formation.^18^ However, many patients already have substantial amyloid deposition at diagnosis, and these interventions are less effective against established fibrils. Thus, agents capable of directly disrupting pre-existing TTR amyloid—so-called “amyloid disruptors”—are urgently needed. Previously, we developed a cell-based high-throughput screening method to directly examine TTR amyloid fibril formation at neutral pH.^19^ Moreover, we identified plant- and marine sponge-derived naphthoquinone analogues that effectively disrupt pre-formed amyloid fibrils.^20^

To address the limited efficacy of current therapies in patients with a high TTR amyloid burden, this study aimed to identify TTR-selective amyloid disruptors of established fibrils and to define structural features underpinning this activity. Accordingly, we screened plant extracts, prioritized hits by potency and selectivity, and evaluated the candidate compound 1,2,3,4,6-penta-*O*-galloyl-β-D-glucose (PGG) using *in vitro* fibril assays, an *in vivo Caenorhabditis elegans* model expressing human TTR fragments, and patient-derived cardiac TTR fibrils. These findings addressed the unmet need for interventions effective in advanced disease.

## Results

### Pomegranate leaf-and-branch extracts disrupt V30M TTR amyloid fibrils

**Figure 1:**
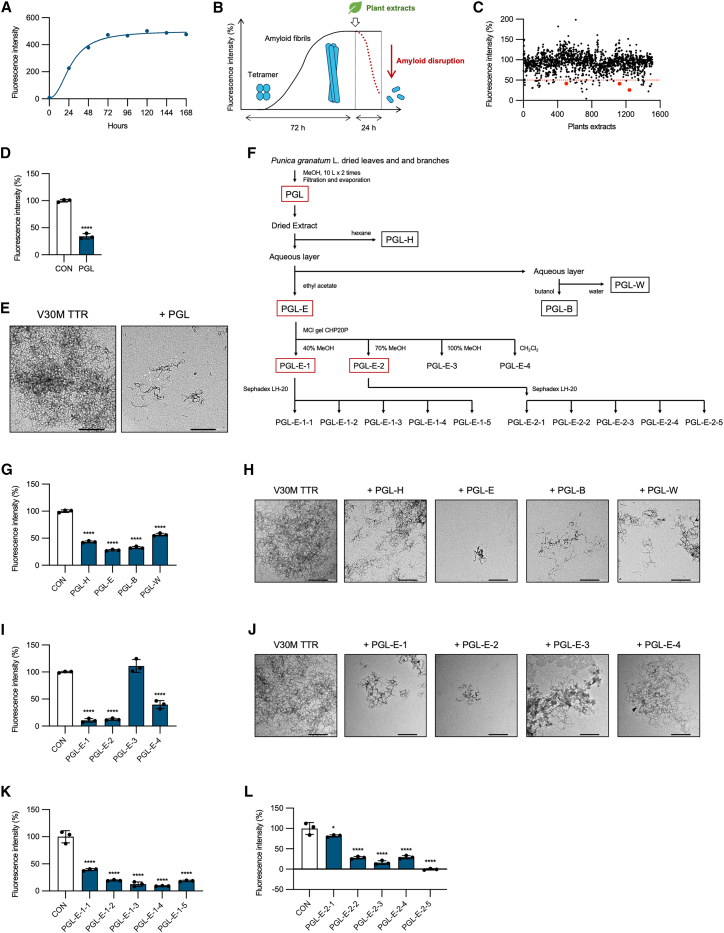
Identification of PGL with TTR amyloid-disrupting activity (A) Time course of V30M TTR amyloid fibril formation monitored by thioflavin T (Th-T) fluorescence. (B) Schematic of the amyloid-disruption assay. V30M TTR tetramers were incubated in acetate buffer (pH 4.4) at 37°C for 72 h to form amyloid fibrils. Plant extracts were then added, and samples were incubated for an additional 24 h at 37°C before analysis by Th-T fluorescence assay. Fluorescence intensity is shown as a percentage relative to untreated controls. (C) Th-T assay screening of 1,509 extracts from various plant parts. Red dots indicate extracts from pomegranate branches, flowers, and leaves. Hits were defined as ≤50% of the untreated control signal. (D) V30M TTR amyloid fibrils treated with 0.5 mg/mL PGL for 24 h and analyzed by Th-T assay. (E) TEM images of V30M TTR fibrils following 24 h treatment with PGL. (F) Fractionation scheme of PGL. (G and I) V30M TTR amyloid fibrils were incubated for 24 h with 0.5 mg/mL of (G) the hexane (PGL-H), ethyl acetate (PGL-E), butanol (PGL-B), and water (PGL-W) fractions, or (I) subfractions of PGL-E, followed by Th-T fluorescence analysis. (H and J) TEM images corresponding to (G) and (I). (K and L) V30M TTR amyloid fibrils were incubated for 24 h with 0.5 mg/mL of further subfractions derived from (K) PGL-E−1 and (L) PGL-E−2, followed by Th-T assay. Data of Th-T assay represent mean ± SD of technical triplicates. Statistical significance was determined using Student’s *t* test or Dunnett’s test (∗*p* < 0.05, ∗∗∗∗*p* < 0.0001 vs. untreated control).

To identify plant extracts capable of disrupting amyloid fibrils, we performed a thioflavin T (Th-T) binding assay, which quantitatively measures amyloid fibril formation. While most previous studies have identified TTR tetramer stabilizers by simultaneously treating TTR tetramers with candidate compounds and assessing amyloid fibril formation using Th-T assays, our study employed a distinct approach. We first incubated V30M TTR tetramers at pH 4.4°C and 37°C for 0–168 h to promote amyloid fibril formation. After 72 h of incubation, amyloid formation reached a plateau (Figure 1A). Therefore, we established a screening protocol in which amyloid fibril formation was induced for 72 h, followed by treatment of the pre-formed fibrils with plant extracts and evaluation using the Th-T assay (Figure 1B). V30M TTR tetramers were incubated at pH 4.4°C and 37°C for 72 h to promote amyloid fibril formation. We then screened 1,509 plant extracts from Kumamoto University’s natural product library (Useful and Unique Natural Products for Drug Discovery, UpRod), which was also utilized in our previous study.^21^ After incubation with plant extracts for 24 h, amyloid fibril formation was assessed. Among these extracts, 49 were identified as hits, defined as samples showing Th-T fluorescence intensity below 50% of the control (set as 100%). Notably, among these hits, only pomegranate (*Punica granatum* L.) extracts demonstrated activity across multiple plant parts, including branches, flowers, and leaves (Figure 1C). A concentration of 0.5 mg/mL of pomegranate leaf-and-branch extracts (PGL) effectively disrupted pre-formed 0.2 mg/mL V30M TTR amyloid fibrils, resulting in a ∼65.8% reduction in Th-T fluorescence intensity compared with the control (Hedges’ g = −13.6 [95.0% confidence interval {CI} −29.4, −11.7]), indicating a strong fibril-disrupting effect (Figure 1D and Table S1). Transmission electron microscopy (TEM) imaging further confirmed fibril disruption, revealing fragmented fibers in PGL-treated samples compared to untreated controls (Figure 1E). Unless otherwise indicated, extracts, fractions, and pure compounds were dosed from DMSO stocks into aqueous assay buffer such that the final DMSO was 1% (v/v) in all wells; controls were vehicle-matched, fibril-only samples.

### Bioactivity-guided fractionation identifies active fractions disrupting TTR amyloid fibrils from pomegranate extracts

To elucidate the active compounds responsible for the amyloid-disrupting activity observed in PGL, we first fractionated PGL using solvents of increasing polarity: hexane, ethyl acetate, *n*-butanol, and water, yielding fractions designated as PGL-H, PGL-E, PGL-B, and PGL-W, respectively (Figure 1F). The amyloid-disrupting activities of these fractions were assessed against pre-formed 0.2 mg/mL V30M TTR amyloid fibrils at a concentration of 0.5 mg/mL using both Th-T assays and TEM. All fractions exhibited some degree of activity; however, PGL-E showed the highest potency, markedly reducing Th-T fluorescence intensity by approximately 72.1% compared to the control (Hedges’ g = −30.2 [95% CI: −37.3, −26.9]), indicating strong disruption of amyloid fibrils (Figure 1G and 1H, and Table S1). PGL-E was then subjected to MCI gel CHP20P column chromatography to obtain four sub-fractions, PGL-E−1 to PGL-E−4 (Figure 1F). Th-T assays and TEM indicated that fractions PGL-E−1 and PGL-E−2 markedly reduced Th-T fluorescence intensity by approximately 89.5% and 87.4%, respectively, compared to the control (PGL-E−1, Hedges’ g = −27.4 [95% CI: −54.5, −23.6]; PGL-E−2, Hedges’ g = −40.7 [95 % CI: −63.1, −37.3]), exhibiting robust amyloid-disrupting activity (Figures 1I–1J and Table S1).

Subsequently, fractions PGL-E−1 and PGL-E−2 were subjected to additional separation and purification using Sephadex LH-20 column chromatography, yielding fractions PGL-E−1-1 to PGL-E−1-5 and PGL-E−2-1 to PGL-E−2-5, respectively (Figure 1F). Th-T assays revealed strong amyloid-disrupting activity in fractions PGL-E−1-2 to PGL-E−1-4 and PGL-E−2-2 to PGL-E−2-4, reflected by large negative effect sizes (Hedges’ g): PGL-E−1-2, −8.15 (95% CI: −36.0, −6.83); PGL-E−1-3, −8.32 (−21.7, −6.94); PGL-E−1-4, −9.23 (−40.3, −7.85); PGL-E−2-2, −5.48 (−16.7, −4.31); PGL-E−2-3, −6.23 (−16.5, −4.96); and PGL-E−2-4, −5.32 (−15.3, −4.16) (Figure 1K, 1L, and Table S1). Although PGL-E−1-5 and PGL-E−2-5 also appeared active, they were excluded from further analysis due to potential dye interference with Th-T fluorescence (Figure 1K and 1L).

### 1,2,3,4,6-penta-*O*-galloyl-β-glucose (PGG) was identified as a TTR-specific amyloid disruptor

**Figure 2:**
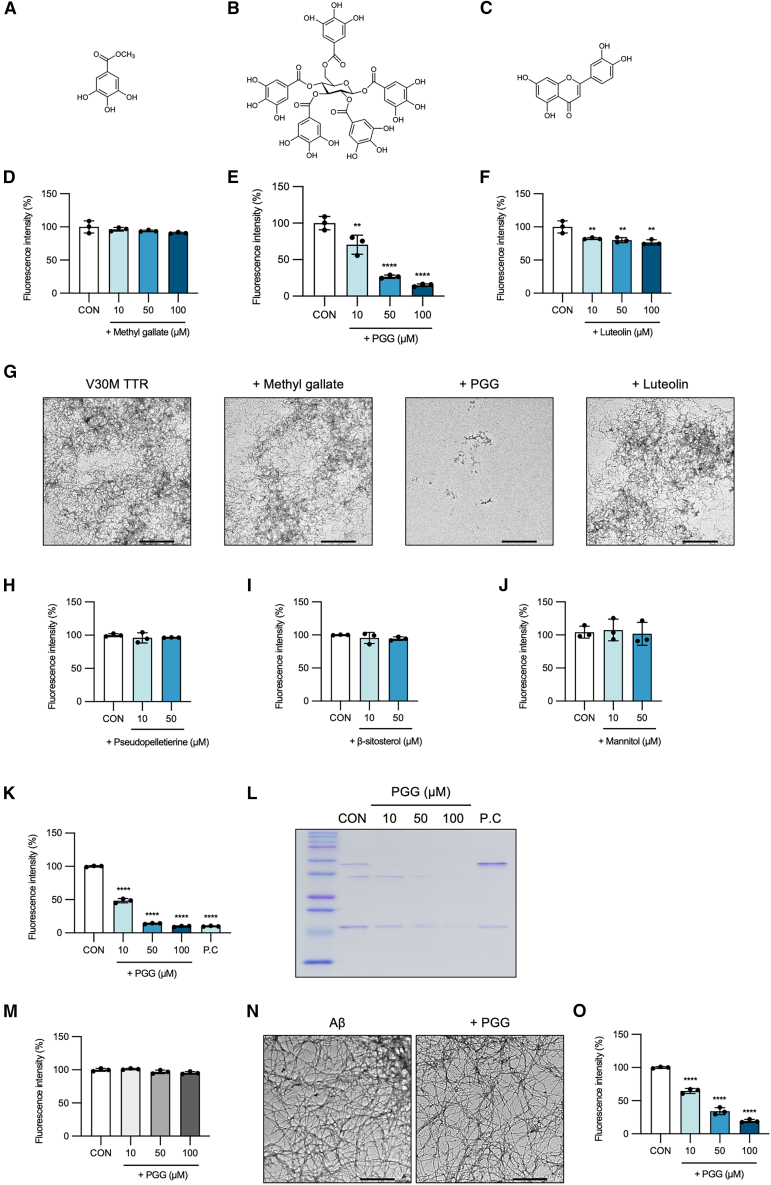
1,2,3,4,6-penta-*O*-galloyl-β-glucose selectively disrupts TTR amyloid fibrils (A–C) Chemical structures of isolated compounds from certain subfractions. (D–F) V30M TTR amyloid fibrils were incubated for 24 h with the indicated concentrations of methyl gallate, PGG, or luteolin and analyzed using the Th-T fluorescence assay. (G) TEM images of V30M TTR fibrils treated with 100 μM of each compound. (H–J) V30M TTR amyloid fibrils were incubated for 24 h with 10 or 50 μM of three representative pomegranate constituents (pseudopelletierine, β-sitosterol, and mannitol) and analyzed by the Th-T fluorescence assay. (K) V30M TTR tetramers were incubated for 72 h with the indicated concentrations of PGG and analyzed by the Th-T fluorescence assay to evaluate inhibition of fibril formation. (L) Monomeric, dimeric, and tetrameric V30 M TTR species in the samples from (K) were assessed by SDS-PAGE followed by CBB staining. (M) Aβ_1_–_42_ was incubated in Tris-HCl buffer (pH 7.4) at 37°C for 24 h to form fibrils, followed by treatment with 10, 50, or 100 μM PGG for an additional 24 h, and analyzed by the Th-T fluorescence assay. (N) TEM images of Aβ_1–42_ fibrils after treatment with 100 μM PGG. (O) Monomeric V30M TTR (V30M M-TTR) was incubated in HEPES buffer (pH 7.4) at 37°C for 24 h to form amyloid fibrils, followed by treatment with 10, 50, or 100 μM PGG for an additional 24 h. Data from the Th-T assay represent mean ± SD of technical triplicates. Statistical significance was determined using Dunnett’s test (∗∗*p* < 0.01, ∗∗∗∗*p* < 0.0001 vs. control).

Using nuclear magnetic resonance (NMR) analysis, we identified three candidate active compounds—methyl gallate,^22^ 1,2,3,4,6-penta-*O*-galloyl-β-glucose (PGG),^23^ and luteolin^24^—from fractions PGL-E−1-1, PGL-E−1-4, and PGL-E−2-2, respectively (Figures 1F; 2A–2C, and S1–S3). Subsequent Th-T assays at concentrations of 10, 50, and 100 μM demonstrated concentration-dependent amyloid-disrupting activity of PGG against V30M TTR fibrils, with Th-T fluorescence intensity markedly reduced by 29.5, 73.4, and 85.0% compared to the control, corresponding to Hedges’ g values of −2.08 (95% CI: 2.96, −1.45), −8.84 (−17.9, −7.24), and −10.3 (−20.4, −8.6), respectively (Figures 2D–2F and Table S1). TEM imaging further confirmed that 100 μM PGG induced notable fragmentation of V30M TTR amyloid fibrils (Figure 2G). In contrast, other compounds commonly present in pomegranate root bark and seeds, such as pseudopelletierine, β-sitosterol, and mannitol, did not reduce Th-T fluorescence intensity (Figures 2H–2J).

Consistent with the previously reported inhibitory effects of polyphenols on TTR amyloid fibril formation,^18^ we confirmed that PGG exhibits this activity. V30M TTR tetramers were incubated with 10, 50, and 100 μM PGG at pH 4.4°C and 37°C for 72 h. PGG inhibited V30M TTR amyloid fibril formation in a concentration-dependent manner. PGG at 10, 50, and 100 μM reduced the Th-T fluorescence intensity by 51.7%, 86.0%, and 90.2%, respectively, corresponding to Hedges’ g values of −17.2 (95.% CI: −35.7, −14.6), −78.9 (−114, −71.4), and −82.3 (−124, −76.4). We used diflunisal (10 μM), a known TTR tetramer stabilizer, as a positive control (Figure 2K and Table S1). Assessment of TTR tetramer stabilization by Coomassie Brilliant Blue (CBB) staining revealed that PGG did not stabilize TTR tetramers, suggesting that its inhibitory effect on amyloid formation occurs via a breakage or disruptor mechanism (Figure 2L).

To assess the specificity of PGG, we investigated its effect on amyloid fibrils formed by the Alzheimer’s disease (AD)-related peptide amyloid β_1–42_ (Aβ_1–42_). Amyloid fibrils of Aβ_1–42_ were prepared by incubation at pH 7.4°C and 37°C for 24 h, followed by treatment with 10, 50, or 100 μM of PGG for an additional 24 h. Interestingly, under these conditions, PGG did not reduce Th-T fluorescence intensity, indicating that it failed to disrupt Aβ_1–42_ amyloid fibrils (Figures 2M and 2N).

Given that V30M TTR amyloid fibrils form under acidic conditions, we examined whether the amyloid-disrupting activity of PGG was dependent on pH. To facilitate amyloid formation under neutral conditions, we utilized recombinant V30M monomeric TTR (M-TTR), in which mutations F87M/L110M inhibit tetramerization through steric hindrance.^25^^,^^26^ Th-T assays at neutral pH conditions demonstrated that PGG effectively disrupted V30M M-TTR amyloid fibrils, markedly reducing Th-T fluorescence intensity in a concentration-dependent manner by 35.3%, 65.9%, and 80.7% at 10, 50, and 100 μM, respectively, corresponding to Hedges’ g values of −10.4 (95% CI: −25.2, −9.29), −13.7 (−34.1, −11.5), and −33.9 (−65.8, −30.3) (Figure 2O and Table S1). These results indicate that PGG selectively disrupts TTR amyloid fibrils under both acidic and neutral conditions, revealing its potential as a TTR-specific amyloid disruptor.

### Compounds with glycosidic structures bearing galloyl side chains disrupt V30M TTR amyloid fibrils

**Figure 3:**
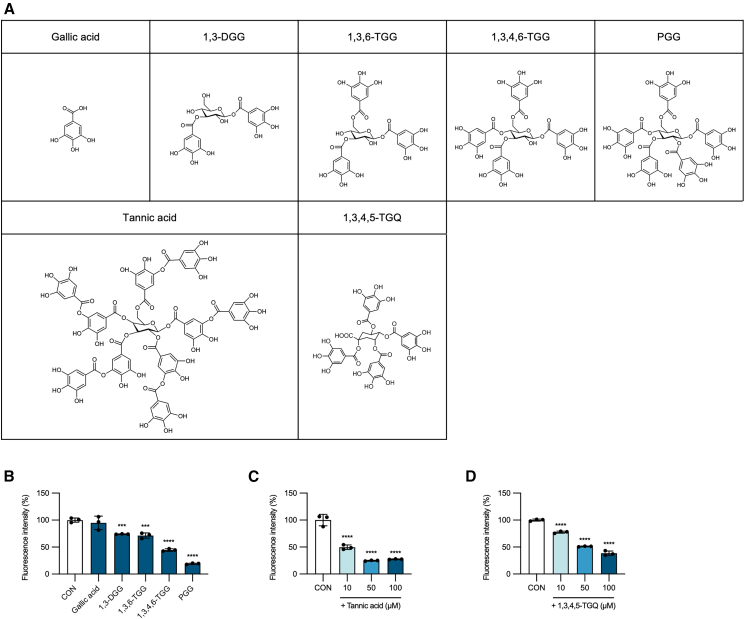
Glycosidic compounds bearing galloyl moieties disrupt V30M TTR amyloid fibrils (A) Chemical structures of glycosidic compounds decorated with galloyl side chains. (B–D) V30M TTR amyloid fibrils were incubated for 24 h with (B) 100 μM or with (C and D) 10, 50, and 100 μM of the indicated compounds and analyzed by the Th-T fluorescence assay. Data represent mean ± SD of technical triplicates. Statistical significance was determined using Dunnett’s test (∗∗∗*p* < 0.001, ∗∗∗∗*p* < 0.0001 vs. control). 1,3-DGG, 1,3-di-*O*-galloyl-β-D-glucose; 1,3,6-TGG, 1,3,6-tri-*O*-galloyl-β-D-glucose; 1,3,4,6-TGG, 1,3,4,6-tetra-*O*-galloyl-β-D-glucose; 1,3,4,5-TGQ, 1,3,4,5-tetra-*O*-galloyl quinic acid.

Next, we focused on the structural characteristics of PGG, specifically its glycosidic core decorated with five galloyl side chains. To explore structure-activity relationships, we evaluated the amyloid-disrupting activity of structurally related glycosidic compounds bearing different numbers of galloyl groups: 1,3-DGG (1,3-di-*O*-galloyl-β-D-glucose), 1,3,6-TGG (1,3,6-tri-*O*-galloyl-β-D-glucose), and 1,3,4,6-TGG (1,3,4,6-Tetra-*O*-galloyl-β-D-glucose) (Figure 3A). Remarkably, we observed that the disruption of V30M TTR amyloid fibrils increased proportionally with the number of galloyl side chains (Figure 3B). Furthermore, we evaluated tannic acid, a glycosidic compound containing ten galloyl side chains that is widely used in antidiarrheal agents. Tannic acid at 10, 50, and 100 μM reduced Th-T fluorescence intensity by 50.4%, 75.2%, and 72.5%, respectively, compared with the control, corresponding to Hedges’ g values of −5.0 (95.0%CI 27.0, −4.05), −8.01 (−134, −7.13), and −7.73 (−137, −6.84), demonstrating potent amyloid-disrupting activity against V30M TTR fibrils (Figure 3C and Table S1). Additionally, 1,3,4,5-tetra-*O*-galloyl quinic acid (1,3,4,5-TGQ), a quinic acid derivative containing four galloyl groups, also exhibited significant V30M TTR fibril-disrupting activity. At concentrations of 10, 50, and 100 μM, 1,3,4,5-TGQ reduced Th-T fluorescence intensity by 22.3%, 48.8%, and 61.5%, respectively, compared with the control, corresponding to Hedges’ g values of −9.67 (95.0%CI −13.1, −8.33), −29.5 (−70.2, −26.2), and −15.4 (−32.1, −13.3) (Figure 3D and Table S1). Collectively, these findings indicate that attachment of galloyl groups to a core ring structure is essential for TTR amyloid-disrupting activity, with efficacy increasing as the number of galloyl groups increases.

### PGL disrupts WT TTR amyloid fibrils and Aβ_1–42_ amyloid fibrils

After evaluating the amyloid-disrupting effects of PGL and its derivatives on V30M TTR fibrils associated with ATTRv amyloidosis, we next explored their potential therapeutic activity against age-related ATTRwt amyloidosis and AD.

**Figure 4:**
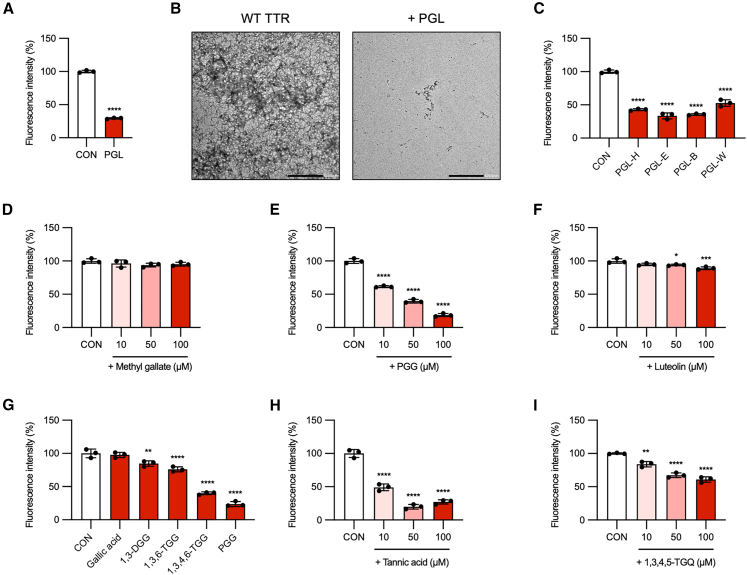
PGG and glycosidic compounds bearing galloyl moieties disrupt WT TTR amyloid fibrils (A) Wild-type (WT) TTR tetramers were incubated in acetate buffer (pH 3.8) at 37°C for 72 h to induce amyloid fibril formation, then treated with 0.5 mg/mL PGL for 24 h and analyzed by Th-T fluorescence assay. (B) TEM images corresponding to (A). (C) WT TTR fibrils were treated for 24 h with 0.5 mg/mL of PGL-H, PGL-E, PGL-B, or PGLW fractions and analyzed by Th-T assay. (D–F) WT TTR fibrils were incubated for 24 h with 10, 50, or 100 μM of three compounds isolated from PGL and analyzed by Th-T assay. (G–I) WT TTR fibrils were treated for 24 h with glycosidic compounds bearing galloyl side chains at 100 μM (G) or 10, 50, and 100 μM (H and I), followed by Th-T assay. Data of Th-T assay represent mean ± SD of technical triplicates. Statistical significance was determined using Student’s *t* test (A) (∗∗∗∗*p* < 0.0001 vs. control) and Dunnett’s test (C–I) (∗*p* < 0.05, ∗∗*p* < 0.01, ∗∗∗∗*p* < 0.0001 vs. control).

Initially, we assessed the effect of PGL on WT TTR amyloid fibrils. WT TTR tetramers (0.2 mg/mL) were incubated at pH 3.8°C and 37°C for 72 h to induce fibril formation, followed by treatment with 0.5 mg/mL PGL for 24 h. PGL reduced Th-T fluorescence intensity to 29.5% of the control (set as 100%), corresponding to a 70.5% decrease (Hedges’ g = −38.8 [95.0%CI −82.0, −34.0]), indicating that PGL disrupted preformed WT TTR amyloid fibrils, similar to its effect on V30M TTR fibrils (Figures 4A, 4B and Table S1). Among the PGL fractions, PGL-E reduced the Th-T fluorescence intensity of WT TTR amyloid fibrils similar to its effect on V30M TTR fibrils. Specifically, Th-T fluorescence was reduced by 66.5% (Hedges’ g = −14.6 [95.0%CI −16.0, −13.8]) (Figure 4C and Table S1). A similar trend was observed for the isolated compounds: PGG at 10, 50, and 100 μM reduced Th-T fluorescence in a dose-dependent manner by 38.5%, 60.5%, and 81.3%, respectively, with corresponding Hedges’ g values of −11.5 (95.0%CI −28.5, −9.75), −15.3 (−22.9, −13.1), −21.7 (−35.0, −18.7) (Figures 4D–4F and Table S1). We also evaluated structurally related galloyl-containing compounds, confirming that activity increased with an increasing number of galloyl groups, a pattern also observed for tannic acid and 1,3,4,5-TGQ (Figures 4G–4I). These results reaffirm the critical role of galloyl substituents in conferring TTR amyloid-disrupting activity.

Subsequently, we examined the potential of PGL to disrupt amyloid fibrils formed by the AD-related peptide Aβ_1–42_. Aβ_1–42_ fibrils (10 μM) were generated by incubating peptides at pH 7.4 and 37°C for 24 h, followed by exposure to 0.5 mg/mL PGL for an additional 24 h^20^. PGL reduced Th-T fluorescence intensity by 57.9% (Hedges’ g = −14.7 [95.0%CI −25.4, −12.7]), indicating effective disruption of Aβ_1–42_ fibrils (Figures S4A, S4B, and Table S1). Notably, fractionation analysis indicated that the highest Aβ_1–42_ amyloid-disrupting activity was observed in the PGL-H fraction, followed by PGL-E, PGL-B, and PGL-W. PGL-H reduced Th-T fluorescence intensity by 70.8% (Hedges’ g = −7.84 [95.0%CI −19.0, −6.38]), indicating potent disruption of Aβ_1–42_ fibrils (Figure S4C). Interestingly, PGG, which disrupted TTR fibrils, did not affect Aβ_1–42_ fibrils (Figures 2M and 2N), suggesting that distinct components within PGL are responsible for disrupting Aβ_1–42_ amyloid fibrils.

### PGG disrupts TTR_81–127_ deposits and extends lifespan and healthspan in nematodes

Having demonstrated amyloid disruption *in vitro*, we next evaluated the *in vivo* efficacy of PGG isolated from PGL. Because the existing TTR transgenic mouse and rat models do not consistently manifest phenotypes reflective of ATTR amyloidosis toxicity,^27^^,^^28^ we used transgenic nematodes (TTR_81–127_ nematodes) for rapid, organism-level evaluation of the biological effects of compounds. This nematode model expresses a fusion protein of the TTR_81–127_ fragment, a highly amyloidogenic C-terminal fragment of TTR,^19^ and EGFP in body wall muscle cells, leading to increased oxidative stress, impaired motility, and reduced lifespan.^29^

**Figure 5:**
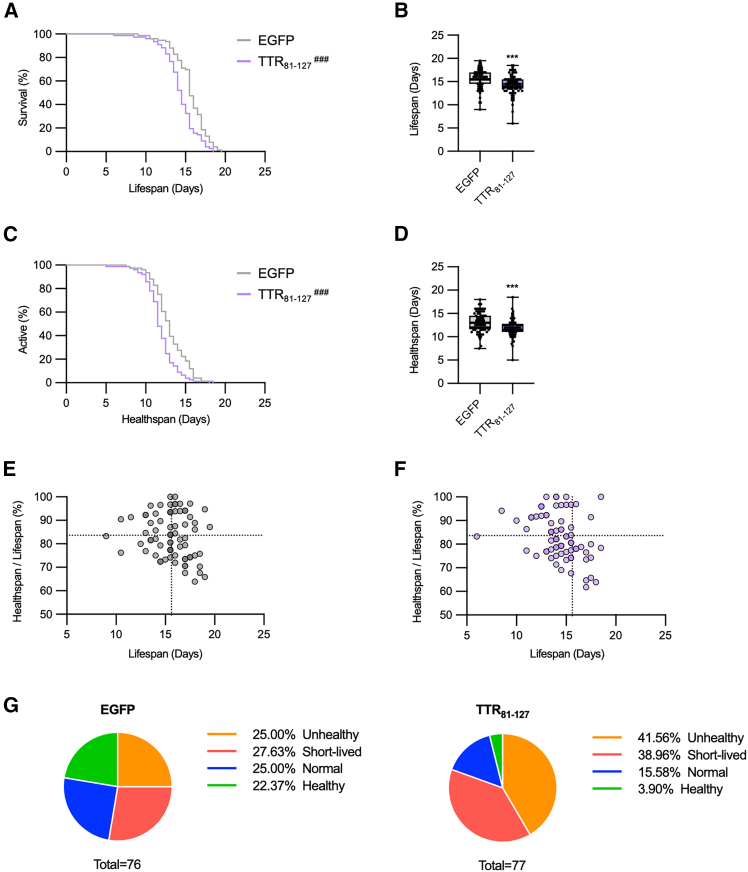
TTR_81–127_ expression shortens lifespan and healthspan in *C. elegans* (A and C) Lifespan (A) and healthspan (C) curves of EGFP-expressing nematodes (*n* = 76) and TTR_81–127_-expressing nematodes (*n* = 77). (B and D) Boxplots showing lifespan (B) and healthspan (D) from three independent biological replicates. (E and F) Scatterplots of individual lifespan versus healthspan-to-lifespan ratio (%). Dashed lines represent the mean values for the EGFP control group along the *x* and *y* axes. (G) Pie charts showing the distribution of nematodes across defined lifespan/healthspan categories. Graphs are representative of three independent experiments. Data are presented as mean ± SD. Statistical significance was assessed using the log-rank test (A and C) (^###^*p* < 0.001 vs. EGFP) and Student’s *t* test (B and D) (∗∗∗*p* < 0.001 vs. EGFP).

Initially, we validated the TTR_81–127_ nematode model by evaluating their lifespan and healthspan using *C. elegans* healthspan auto-monitoring system (C-HAS).^30^^,^^31^^,^^32^^,^^33^ C-HAS provides objective measurements by capturing nematode movement every 12 h. Healthspan was defined as the period during which worms maintained active movement, while lifespan was determined by cessation of both movement and body orientation changes. Consistent with previous reports,^29^ TTR_81–127_ nematodes exhibited shorter lifespans compared to control EGFP nematodes. Healthspan, as determined by C-HAS, was also significantly reduced in TTR_81–127_ nematodes (Figures 5A–5D). Moreover, intrapopulation analysis indicated a marked decline in “healthy” individuals (those with both long lifespan and high healthspan) from 22.37% in EGFP nematodes to 3.9% in TTR_81–127_ nematodes. Conversely, the proportion of short-lived and “unhealthy” individuals increased from 52.63% to 80.52% (Figures 5E–5G).

**Figure 6:**
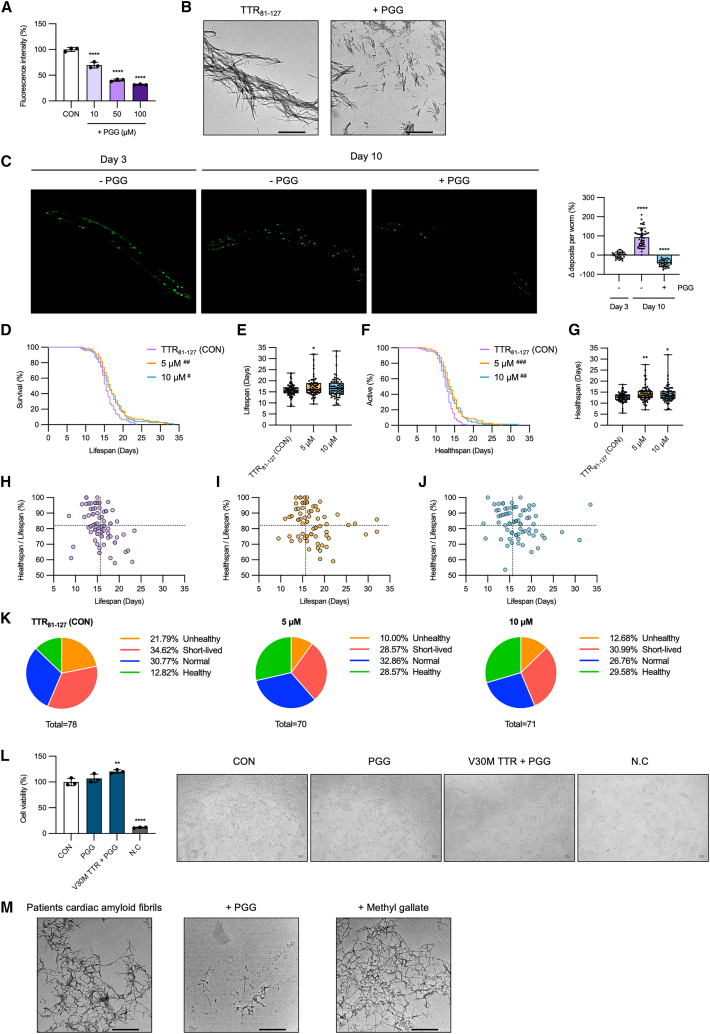
PGG reduces TTR_81–127_ deposits and extends lifespan and healthspan in *C. elegans* while disaggregating patient-derived cardiac TTR amyloid fibrils (A) The TTR_81–127_ fragment was incubated in 1× PBS (pH 7.4) at 37°C for 24 h to form amyloid fibrils, then treated with 10, 50, or 100 μM PGG for 24 h. Samples were analyzed by Th-T assay. (B) TEM images of TTR_81–127_ fibrils after 24 h incubation with 100 μM PGG. (C) Fluorescence images of TTR deposits in 10-day-old TTR_81–127_ nematodes treated with 10 μM PGG for 7 days. Control: 3-day-old untreated nematodes. Right: quantification of TTR deposits. (D–G) Lifespan (D) and healthspan (F) curves of TTR_81–127_ nematodes treated with 5 or 10 μM PGG (control, *n* = 78; 5 μM, *n* = 77; 10 μM, *n* = 71). (E–G) Boxplots summarizing lifespan (E) and healthspan (G) from three independent biological replicates. (H–J) Scatterplot showing the relationship between individual lifespan and healthspan-to-lifespan ratio (%). Dashed lines represent mean values of the control group along each axis. (K) Pie charts showing the distribution of nematodes across defined lifespan/healthspan categories. (L) SH-SY5Y cells were treated for 24 h with PGG alone or with the PGG-generated degradation-product preparation and then incubated with CCK-8 for 3 h and analyzed at an absorbance of 450 nm. Viability was normalized to buffer-only/nontreated cells (100%). 1% Triton X-100 served as a lytic control. (M) TEM images of patient-derived cardiac TTR amyloid fibrils following 24 h incubation with 100 μM PGG. Graphs are representative of three independent experiments. Data are presented as mean ± SD. Statistical significance was determined using the log-rank test for survival and healthspan curves (D and F) (^#^*p* < 0.05, ^##^*p* < 0.01, ^###^*p* < 0.001 vs. control) and Dunnett’s test for other comparisons (A, B, E, and G) (∗*p* < 0.05, ∗∗*p* < 0.01, ∗∗∗∗*p* < 0.0001 vs. control).

We hypothesized that PGG treatment might reduce TTR deposits and extend both lifespan and healthspan. We first examined whether the TTR_81–127_ fragment, a highly amyloidogenic C-terminal fragment of TTR capable of forming amyloid fibrils at neutral pH,^19^ is disrupted by 10, 50, and 100 μM PGG treatments. PGG reduced Th-T fluorescence intensity by 30.3%, 59.5%, and 68.3% at 10, 50, and 100 μM, respectively (10 μM: Hedges’ g = −4.99 [95.0%CI −8.75, −3.85]; 50 μM: Hedges’ g = −15.0 [−27.3, −13.8]; 100 μM: Hedges’ g = −19.1 [88.1, −17.8]), indicating effective disruption of TTR_81–127_ fragment amyloid fibrils (Figures 6A, 6B, and Table S1). We then cultured TTR_81–127_ nematodes from the L4 stage on nematode growth medium (NGM) plates containing 10 μM PGG for 7 days and assessed TTR deposits. Compared to untreated 3-day-old TTR_81–127_ nematodes, set as 0%, untreated 10-day-old nematodes showed a 93.7% increase in TTR deposits, whereas the PGG-treated group exhibited a 42.9% decrease in TTR deposits after 7 days (Figure 6C), indicating that PGG effectively disrupts TTR aggregates *in vivo*.

Subsequently, we used C-HAS to evaluate whether reduced TTR deposits correlate with improved lifespan and healthspan. TTR_81–127_ nematodes exposed to 5 and 10 μM PGG showed significantly extended lifespans compared to control groups (Figure 6D). Healthspan analysis similarly revealed enhanced active movement patterns in PGG-treated worms (Figure 6F). Quantitative analyses confirmed significant increases in both lifespan and healthspan with PGG treatment (Figures 6E and 6G). Additionally, intrapopulation analysis demonstrated that PGG treatment shifted worm populations toward healthier aging profiles: the proportion of worms classified as “healthy” increased from 12.82% to 28.57% (5 μM) and 29.58% (10 μM), while “unhealthy” individuals decreased from 21.79% to 10% (5 μM) and 12.68% (10 μM) (Figures 6H–6K).

To assess whether PGG-generated degradation products are cytotoxic, we exposed SH-SY5Y cells to recombinant V30M-TTR amyloid fibrils disrupted by PGG and measured cell viability. Values were normalized to the buffer-only/nontreated control (=100%). PGG alone yielded ∼106 ± 6% viability, and the degradation-product preparation yielded ∼118 ± 6%, showing no reduction relative to buffer-only or nontreated cells. To set the assay’s lower bound and verify that cytotoxicity is detectable, we included 1% Triton X-100 as a lytic (“dead-cell”) control, which reduced viability to ∼15 ± 4%. These results indicate that the PGG-generated degradation products are not cytotoxic under our conditions (Figure 6L).

To investigate whether the observed improvements were specifically due to amyloid disruption, we tested the effects of PGG in EGFP nematodes without TTR expression. In these nematodes, exposure to 5 and 10 μM PGG did not extend lifespan or healthspan compared to untreated controls (Figures S5A–S5D). Intrapopulation analysis also indicated no beneficial shift in aging profiles with PGG treatment (Figures 6E–6H). These findings collectively suggest that the beneficial effects of PGG on lifespan and healthspan specifically result from its capacity to disrupt TTR amyloid deposits.

### PGG disrupts TTR amyloid fibrils isolated from patient cardiac tissue samples

Having demonstrated the amyloid-disrupting activity of PGG in both *in vitro* and *in vivo* models, we next sought to confirm its efficacy against human-derived amyloid fibrils. We tested amyloid fibrils isolated from the cardiac tissue of a patient diagnosed with ATTRv amyloidosis. The patient-derived cardiac amyloid fibrils were treated with 100 μM PGG for 24 h and subsequently evaluated using TEM. Our results clearly showed that PGG significantly disrupted the patient’s cardiac amyloid fibrils *ex vivo*. In contrast, methyl gallate, which exhibited no amyloid-disrupting activity *in vitro*, similarly showed no effect in this *ex vivo* assay (Figure 6M). These findings strongly suggest that PGG effectively disrupts not only amyloid fibrils generated *in vitro* but also amyloid fibrils isolated directly from patients.

## Discussion

This study identified pomegranate-derived PGG through plant-based screening as a natural-product-derived amyloid disruptor with activity against TTR amyloid. Pomegranate is a deciduous tree species primarily known for its fruit. Originally native to the Middle East, it is now widely cultivated throughout the Mediterranean region, Asia, and North Africa.^34^ Historically valued as food and medicine, pomegranate has demonstrated diverse pharmacological activities, including antidiabetic,^35^ antitumor,^36^ anti-inflammatory,^36^ and antifungal effects.^37^ While previous research predominantly focused on the fruit, other plant parts—such as leaves, branches, and seeds—remain relatively underexplored. Further investigation of these underutilized components could reveal additional therapeutic compounds.

Through targeted screening of natural products, this study identified PGG,^38^ a hydrolyzable tannin abundant in pomegranate leaves and branches, as a potent and selective TTR amyloid disruptor. Previous *in vitro* and limited *in vivo* studies have described multiple beneficial properties of PGG, including antidiabetic, antitumor, anti-inflammatory, and antiviral activities, supporting its potential as a bioactive scaffold.^38^^,^^39^ *In vitro* assays demonstrated that PGG robustly disrupted multiple types of TTR amyloid fibrils (V30M, WT, M-V30M, and TTR_81–127_) without affecting amyloid fibrils composed of Aβ_1–42_, indicating target selectivity for TTR amyloids under the tested conditions.

PGG disaggregated TTR fibrils but not Aβ_1-42_ under both mildly acidic and neutral conditions, suggesting that selectivity reflects fibril surface chemistry and topology rather than a single obligate pathway. Patient-derived TTR fibrils present solvent-exposed basic patches and accessible aromatic side chains (e.g., Y116) along surface grooves,^40^ whereas Aβ_1–42_ fibrils display a distinct electrostatic landscape with fewer accessible cationic sites and many aromatic residues buried within the cross-β core.^41^ In keeping with precedents for polyphenols—most notably EGCG, which remodels tau fibrils by engaging solvent-exposed grooves through multivalent π-π and hydrogen-bonding contacts^42^—we hypothesize that PGG’s multiple galloyl rings and hydroxyl groups similarly engage TTR surfaces. pH likely modulates these interactions: at lower pH, increased protonation of fibril side chains can alter salt bridge networks and enhance local cationic character, favoring PGG encounter and binding; at neutral pH, although PGG, which lacks a free carboxylate and is only weakly deprotonated at phenolic sites, is less influenced by long-range electrostatics, short-range π-π/hydrogen-bond networks and shape complementarity may still permit productive engagement. Although an electrostatic hotspot centered on Arg21 might contribute to binding, the ability of PGG to disaggregate TTR_81–127_ fibrils (which lack Arg21) supports a surface-accessibility model and indicates that Arg21 is not required, albeit it may enhance interactions in full-length fibrils. By contrast, on Aβ surfaces, the limited exposure of complementary aromatic/polar motifs likely impedes analogous interactions across pH. This remains a working model; systematic tests of pH and ionic-strength dependence, pKa/stability assessments of PGG (including potential hydrolysis), and site-directed substitutions (e.g., Y116F and Arg21 variants), coupled with cryo-EM or solid-state NMR, will be needed to define the binding mode and rigorously evaluate these hypotheses.

Interestingly, although PGG itself did not disrupt Aβ_1–42_ fibrils, the parent pomegranate PGL exhibited notable Aβ-disrupting activity. Fractionation studies revealed that this activity primarily resided in a highly lipophilic fraction (PGL-H), suggesting lipid-soluble constituents within PGL-H as promising candidates responsible for Aβ fibril disruption. Identification and characterization of these lipophilic compounds represent valuable directions for future research.

From a translational perspective, the distinction between ATTRv and ATTRwt is important. While significant therapeutic advancements, including liver transplantation, siRNA-based TTR silencing, and tetramer stabilizers targeting specific genetic mutations, have improved outcomes in ATTRv,^15^^,^^16^^,^^17^ therapeutic options for age-related ATTRwt amyloidosis remain limited; tetramer stabilizers have only recently become available.^43^ Although many patients present with a substantial amyloid burden at diagnosis, early detection of ATTRwt amyloidosis remains challenging. In this context, a disruptor that acts on pre-existing deposits could complement stabilizers and silencers. The identification of PGG from underutilized plant components further highlights opportunities for sustainable sourcing.

In conclusion, our data support PGG as a structurally defined, selective disruptor of diverse TTR amyloid fibrils across *in vitro*, *in vivo*, and *ex vivo* systems. While mechanistic details and pharmacokinetics require further elucidation, these findings underscore the therapeutic potential of natural product-based strategies and the value of sustainably sourced, plant-derived compounds as candidates for disease-modifying treatment of ATTR amyloidosis.

### Limitations of the study

Despite its promise, clinical development of PGG faces challenges. Orally administered PGG may undergo gastrointestinal degradation, potentially limiting bioavailability, and its polyphenolic nature raises concerns about nonspecific protein interactions typical of tannins. Nevertheless, the selective disaggregation of TTR versus Aβ and the putative contribution of aromatic/hydrogen-bonding contacts provide a rationale for structure-activity optimization. A further limitation is that our patient-derived evaluation used cardiac tissue from a single V30M donor, which restricts generalizability across patients and genotypes. To probe the mechanism and improve drug-like properties, future studies will (i) assess ionic-strength dependence to evaluate electrostatic contributions; (ii) estimate apparent pKa values and monitor potential hydrolysis to gallic acid under assay conditions by liquid chromatography-mass spectrometry /NMR; (iii) test site-specific contributions (e.g., Y116F and additional aromatic/basic residue substitutions) to sensitivity; (iv) define the binding mode by cryo-EM or solid-state NMR using patient-derived and *in vitro* TTR fibrils; and (v) validate activity in additional samples from multiple V30M patients as well as other pathogenic TTR variants. Collectively, these efforts should clarify whether interactions analogous to those reported for EGCG on tau fibrils^42^ underlie PGG-driven TTR fibril disassembly and guide the design of more selective, bioavailable TTR-specific amyloid disruptors.

## Resource availability

### Lead contact

Further information and requests for resources and reagents should be directed to and will be fulfilled by the lead contact: Tsuyoshi Shuto (tshuto@gpo.kumamoto-u.ac.jp).

### Materials availability

Materials generated in this study, including isolated PGG and plant fractions, are available from the lead contact upon request and completion of a material transfer agreement.

### Data and code availability

• This paper does not report original code.
• Any additional information required to reanalyze the data reported in this paper is available from the lead contact upon request.
• All data reported in this paper are available from the lead contact upon reasonable request.

## Acknowledgments

This work was supported by the Useful and Unique Natural Products for Drug Discovery and Development (UpRod), the Program for Building Regional Innovation Ecosystems at Kumamoto University, the Health Life Science S-HIGO (Health life science: Interdisciplinary and Glocal Oriented) Professional Fellowship Program, and the Program for Fostering Innovators to Lead a Better Co-being Society (grant no. JPMJSP2127) (MEXT, Japan). We acknowledge Mr. Masato Watanabe, Technical Specialist at the Kumamoto University Medicinal Plant Garden, for his assistance with plant harvesting.

## Author contributions

A.K., H.K., and T. Shuto designed research. A.K., N.H., R.S., Y.F., H.P.D., S.T., S.Y., and T. Shuto performed research and analyzed data. T.Y., M.M., and T. Sato prepared TTR proteins. A.K., Y.F., K.Y., M.U., and T. Shuto established and utilized *C. elegans* models. H.P.D., S.T., M.W., and S.M. prepared plant extracts. S.Y. and M.U. prepared *ex vivo* samples from patients. A.K., T. Sato, T.N., S.K., and T. Shuto discussed the structure-activity relationship of PGG. A.K., M.A.S., M.M., M.U., T.N., and T. Shuto wrote the paper. H.K., M.U., and T. Shuto supervised the project.

## Declaration of interests

The authors declare no competing interests.

## STAR★Methods

### Key resources table

**Table:** 

REAGENT or RESOURCE	SOURCE	IDENTIFIER
**Bacterial strains**		

*Escherichia coli* OP50	Caenorhabditis Genetics Center (CGC)	Strain OP50
*Escherichia coli* BL21 (DE3)	Merck Millipore Sigma (Novagen)	Cat# 69450
*Escherichia coli* M15	QIAGEN (QIAexpress system)	QIAexpress Data Sheet: E. coli strain M15.

**Biological samples**		

Human cardiac tissue (autopsy), ATTR V30M	This paper; Kumamoto University Human Ethics Review Committee	Approval no. 2651
Pomegranate leaves and branches (*Punica granatum* L.)	Kumamoto University Medicinal Plant Garden	N/A

**Chemicals, peptides, and recombinant proteins**		

Methyl gallate	Tokyo Chemical Industry (TCI, Tokyo, Japan)	Cat#G0017
1,2,3,4,6-penta-*O*-galloyl-β-D-glucose (PGG)	Cayman Chemical (Ann Arbor, MI, USA)	Cat#16007
Luteolin	AdipoGen Life Sciences	Cat#AG-CN2-0098-M025
Pseudopelletierine	Tokyo Chemical Industry (TCI, Tokyo, Japan)	Cat#P2648
β-Sitosterol	Nacalai Tesque, Inc. (Kyoto, Japan)	Cat#31133-94
Mannitol	Nacalai Tesque, Inc. (Kyoto, Japan)	Cat#21302-55
1,3-di-*O*-galloyl-β-D-glucose (1,3-DGG)	Devkota, H.P et al.^44^	N/A
1,3,6-tri-*O*-galloyl-β-D-glucose (1,3,6-TGG)	Devkota, H.P et al.^44^	N/A
1,3,4,6-tetra-*O*-galloyl-β-D-glucose (1,3,4,6-TGG)	Devkota, H.P et al.^44^	N/A
1,3,4,5-Tetra-*O*-galloyl quinic acid (1,3,4,5-TGQ)	Dirar, A.I et al.^45^	N/A
1,4-Naphthoquinone (positive control)	Sasaki, R et al.^20^	N/A
KM001 (negative control)	Sasaki, R et al.^20^	N/A
1,1,1,3,3,3-Hexafluoro-2-propanol (HFIP)	Merck MilliporeSigma	Cat# 804515
Recombinant TTR (V30M)	Miyata, M et al.^46^	N/A
Recombinant TTR (WT)	Miyata, M et al.^46^	N/A
Recombinant TTR (M-V30M)	Sasaki, R et al.^20^ Inada, Y et al.^47^	N/A
Recombinant TTR fragment (TTR_81–127_)	Ueda, M et al.^19^	N/A
Aβ_1–42_ peptide (human)	Peptide Institute Inc. (Osaka, Japan)	Cat#4349-v

**Recombinant DNA**		

Plasmid: pEGFP-1	Takara Bio (formerly Clontech)	Backbone for EGFP fusions
Plasmid: pCKX3305	This paper	Full-length human TTR cloned into pEGFP-1
Plasmid: pCKX3307	This paper	TTR::EGFP in pPD30.38
Plasmid: pCKX3301	This paper	TTR_81–127_::EGFP in pEGFP-1
Plasmid: pPD30.38	A. Fire lab vector	N/A
Plasmid: pCKX3303	This paper	TTR_81–127_::EGFP in pPD30.38
Plasmid: EGFP-only control (pCKX3316)	This paper	EGFP under the unc-54 promoter/enhancer in pPD30.38
Plasmid: pGBHPS (GB1–His tag–HRV3C site expression vector)	This paper	In-house backbone for N-terminal GB1–His fusions with HRV3C cleavage site

**Critical commercial assays**		

Cell Counting Kit-8 (CCK-8)	Dojindo Laboratories (Kumamoto, Japan)	Cat#347-07621

**Experimental models: cell lines**		

SH-SY5Y (human neuroblastoma)	ATCC	CRL-2266

**Experimental models: organisms/strains**		

*C. elegans* transgenic strain expressing human TTR_81–127_	Tsuda, Y et al.^29^	TTR_81-127_::EGFP
*C. elegans* transgenic strain expressing EGFP	Tsuda, Y et al.^29^	TTR::EGFP

**Software and algorithms**		

GraphPad Prism 10	GraphPad Software (San Diego, CA, USA)	https://www.graphpad.com/updates/prism-1000-release-notes
Incucyte® S3 Software	Sartorius (Göttingen, Germany)	https://www.sartorius.com/en/products/live-cell-imaging-analysis/live-cell-analysis-software
BZ-Analyzer	Keyence (Osaka, Japan)	https://www.keyence.com/products/microscope/fluorescence-microscope/bz-x700/models/bz-x710/
DABEST (Estimation Statistics; Web app)	Ho, Y et al.^48^	https://www.estimationstats.com/?utm_source=chatgpt.com#/

**Other**		

JEM-2100 Transmission Electron Microscope	JEOL (Tokyo, Japan)	Engineering Research Equipment Center in Kumamoto University
FP-8500 Spectrofluorometer	JASCO (Tokyo, Japan)	https://www.jasco.co.uk/fp-8500.html
BZ-X710 All-in-One Fluorescence Microscope	Keyence (Osaka, Japan)	https://www.keyence.com/products/microscope/fluorescence-microscope/bz-x700/models/bz-x710/
Carbon-coated copper grids (STEM Cu100P)	Okenshoji Co., Ltd. (Tokyo, Japan)	Cat#10-1012
MCI gel CHP20P	Mitsubishi Chemical Industries Co., Ltd.	75–150 μm
Sephadex LH-20	Amersham Pharmacia Biotech	LH-20
Silica gel	Merck	0.040–0.063 mm
TLC	Merck	Silica gel 60 F254 (0.2 mm)
Useful and Unique Natural Products for Drug Discovery (UpRod) plant extract library (1,509 extracts)	Kumamoto University (UpRod project)	https://uprod-kumamoto.org/en/
Ni-NTA agarose	FUJIFILM Wako Pure Chemical Corporation	Cat# 143-09763
HRV-3C Protease ver.2	FUJIFILM Wako Pure Chemical Corporation	Cat# 312-09591
ize-exclusion column: HiLoad 16/60 Superdex 75 pg	Cytiva (formerly GE Healthcare)	Cat# 28-9893-33

### Experimental model and study participant details

#### Human subjects

Human cardiac tissue (autopsy, ATTR V30M) was obtained under a protocol approved by the Human Ethics Review Committee of Kumamoto University (approval no. 2651), in accordance with the Declaration of Helsinki. Written informed consent was obtained from patients or family members. Tissue was stored at 4°C without fixatives until use; the use of a single donor is acknowledged as a limitation in the discussion.

#### *Caenorhabditis elegans* strains and maintenance

*C. elegans* strains were maintained at 23°C on nematode growth medium (NGM) plates seeded with *Escherichia coli* OP50 following standard procedures.^30^^,^^32^ Transgenic strains expressing EGFP or human TTR_81–127_ were established as described.^29^ Briefly, translational fusions of human TTR to EGFP were built as follows. Full-length TTR was cloned into pEGFP-1 to generate pCKX3305, then subcloned into pPD30.38 under the unc-54 promoter/enhancer to yield pCKX3307 (TTR::EGFP for body-wall muscle expression). A truncated TTR_81–127_::EGFP was generated via pCKX3301 and subcloned into pPD30.38 (pCKX3303); an EGFP-only control plasmid (pCKX3316) was constructed similarly. Plasmid DNA was sequence-verified. Transgenes were introduced into the *C. elegans* germline by standard gonadal microinjection to generate extrachromosomal arrays in the N2 background.

We selected *C. elegans* as the *in vivo* model because it enables live visualization/quantification of proteotoxic aggregates, offers a short lifespan with high-throughput healthspan/lifespan phenotyping, and supports transgenic expression of human TTR fragments that recapitulate aggregation-linked phenotypes at the organismal level, while minimizing ethical burden and cost. In addition, widely adopted mouse ATTR models are lacking, and available rat models exhibit late-onset phenotypes, which limit throughput and extend study timelines. Therefore, *C. elegans* provides a pragmatic platform for screening and mechanistic studies. All *in vivo* experiments involving *C. elegans* were designed and reported in accordance with the ARRIVE 2.0 guidelines (Animal Research: Reporting of *In Vivo* Experiments).

### Method details

#### Purification of the recombinant proteins

Human TTR variants were produced and purified as follows, consolidating previously published procedures. Briefly, ET-22b(+) plasmid with WT or V30M TTR were transformed into *E. coli* BL21(DE3), followed by the induction at OD600 ≈ 0.6 with 1 mM IPTG for 24 h, harvested, sonicated in 20 mM phosphate (pH 7.0), and clarified. TTR variants in the soluble fraction were purified by anion-exchange chromatography followed by reversed-phase HPLC.^46^ pGBHPS-*M*-TTR plasmid with V30M M-TTR was expressed in BL21(DE3) as N-terminal GB1 fusion proteins carrying a His tag and an HRV3C protease site. Overnight seed cultures (2×YT, 100 μg/mL ampicillin, 37°C) were expanded (2×YT, ampicillin) and induced with 0.1 mM IPTG for 24 h at 25°C. Cell pellets were lysed in PBS with 2 mM 2-mercaptoethanol and clarified; proteins were captured on Ni-NTA agarose, tags were removed by HRV3C digestion (4 °C, overnight), and untagged proteins were polished by size-exclusion chromatography (HiLoad 16/60 Superdex 75; 50 mM HEPES, 150 mM NaCl, pH 7.4).^20^^,^^47^ TTR_81–127_ fragment was expressed as a fusion protein with an N-terminal His_6_-tagged full-length WT TTR in *E. coli* M15 cells and purified with Ni-affinity chromatography. The His_6_ tag and TTR tag were cleaved by factor Xa or tobacco etch virus protease, and TTR_81–127_ fragment was purified by reversed-phase HPLC. Those recombinant proteins were dissolved in 0.02% NH_3_ solution, and aliquots were stored at −80°C^19^. Purity of these proteins was monitored by SDS–PAGE and yield by A280.

#### Preparation and aging of amyloid fibrils

Amyloid fibrils were generated by incubating recombinant tetrameric TTRs under established conditions.

• V30M TTR: 200 mM acetate (pH 4.4), 1 mM EDTA, 100 mM KCl, 37°C, 72 h.
• WT TTR: 200 mM acetate (pH 3.8), 1 mM EDTA, 100 mM KCl, 37°C, 72 h.
• M-V30M TTR: HEPES (pH 7.4), 37°C, 72 h.
• TTR_81–127_: 1×PBS (pH 7.4), 37°C, 24 h.

For Aβ_1–42_ fibrils, the lyophilized peptide was pretreated with 1,1,1,3,3,3-hexafluoro-2-propanol (24 h, room temperature), dried under vacuum, dissolved in DMSO (10 μM), diluted with Tris (pH 7.4), and incubated at 37°C for 24 h^20^. All aged fibrils were then co-incubated with test compounds at the indicated concentrations for 24 h before analysis.

#### Plant material, extraction, and fractionation

As listed in the key resources table, we screened a curated library of 1,509 plant extracts from the Useful and Unique Natural Products for Drug Discovery (UpRod) project at Kumamoto University.^21^ For the isolation work described here, dried leaves and branches of pomegranate (*Punica granatum* L.; Kumamoto University Medicinal Plant Garden) were used.

Dried pomegranate leaves and branches (1,000 g) were extracted twice with methanol (10 L). The obtained extract was concentrated under reduced pressure and dried using a rotary evaporator to yield pomegranate extract (PGL, 143.0 g). PGL was partitioned to obtain a hexane-soluble fraction (PGL-H, 4.7 g), an ethyl acetate-soluble fraction (PGL-E, 17.5 g), a butanol-soluble fraction (PGL-B, 60.0 g), and a water-soluble fraction (PGL-W, 54.5 g). PGL-E was dissolved in 40% methanol and subjected to MCI gel CHP20P column chromatography with sequential elution (40% MeOH, 70% MeOH, 100% MeOH, dichloromethane) to afford four fractions (PGL-E-1, 8.1 g; PGL-E-2, 3.3 g; PGL-E-3, 5.4 g; PGL-E-4, 0.9 g). Fraction PGL-E-1 was subjected to Sephadex LH20 column chromatography and eluted with methanol. The eluates were collected in ∼15 ml fractions and analyzed by thin-layer chromatography (TLC). Fractions with similar TLC profiles were pooled to give four subfractions (PGL-E-1-1 to PGL-E-1-4). Subfraction PGL-E-1-1 was further purified by silica gel chromatography, eluting with dichloromethane:methanol:water (8:2:0.1), to obtain methyl gallate (187 mg). Subfraction PGL-E-1-4 was a pure compound and was identified as 1,2,3,4,6-penta-*O*-galloyl-β-glucose (PGG, 200 mg). PGL-E-2 was further purified by Sephadex LH-20 column chromatography (eluted with methanol) followed by silica gel column chromatography (eluted with dicloromethane:methanol:water (8:2:0.1)) to obtain luteolin (48 mg).

#### Spectroscopic characterization of isolated compounds (PGG, methyl gallate, luteolin)

The identities of isolated constituents from the pomegranate ethyl acetate fraction were established by NMR and compared with literature data. For the identification of methyl gallate, the TLC profile with the commercial compound (methyl gallate; TCI, Tokyo, Japan) was also confirmed.

• 1,2,3,4,6-penta-*O*-galloyl-β-D-glucose (PGG)

Identity established by ^1^H/^13^C NMR comparison with the previous report.^23^ NMR assignments follow the numbering shown in Figure S1.

^1^H NMR (600 MHz, CD_3_OD): δ 7.12 (2H, s, H-2‴/6‴), 7.06 (2H, s, H-2′/6′), 6.99 (2H, s, H-2″″/6″″), 6.96 (2H, s, H-2‴′/6‴′), 6.91 (2H, s, H-2″/6″), 6.25 (1H, d, J = 8.0 Hz, H-1), 5.91 (1H, t, H-4), 5.63 (1H, m, H-5), 5.59 (1H, m, H-2), 4.43 (1H, m, H-3), 4.39 (2H, m, H-6).

^13^C NMR (125 MHz, CD_3_OD): δ 93.9 (C-1), 74.1 (C-3), 72.3 (C-4), 69.8 (C-2), 68.8 (C-5), 63.1 (C-6); Galloyl i (1-O-): 119.8 (C-1′), 110.3 (C-2′/6′), 140.0 (C-4′), 146.3 (C-3′/5′), 166.3 (C-7′); Galloyl ii (2-O-): 120.2 (C-1″), 110.4 (C-2″/6″), 140.1 (C-4″), 146.4 (C-3″/5″), 167.0 (C-7″); Galloyl iii (3-O-): 121.1 (C-1‴), 110.7 (C-2‴/6‴), 140.8 (C-4‴), 146.6 (C-3‴/5‴), 168.0 (C-7‴); Galloyl iv (4-O-): 120.3 (C-1″″), 110.5 (C-2″″/6″″), 140.4 (C-4″″), 146.5 (C-3″″/5″″), 167.0 (C-7″″); Galloyl v (6-O-): 120.4 (C-1‴′), 110.5 (C-2‴′/6‴′), 140.4 (C-4‴′), 146.5 (C-3‴′/5‴′), 167.3 (C-7‴′).

• Methyl gallate

Identity established by TLC profile and ^1^H NMR comparison with the previous report.^22^ NMR assignments follow the numbering shown in Figure S2.

^1^H NMR (600 MHz, CD_3_OD): δ 7.06 (2H, s, H-2/H-6), 3.83 (3H, s, OCH_3_).

• Luteolin

Identity established by TLC profile and ^1^H NMR comparison with the previous report.^24^ NMR assignments follow the numbering shown in Figure S3.

^1^H NMR (600 MHz, CD_3_OD) δ 7.41 (2H, m, J = 2.3 Hz, H-2′/H-6′), 6.90 (1H, d, J = 8.3 Hz, H-5′), 6.68 (1H, s, H-3), 6.46 (1H, d, J = 2.2 Hz, H-8), 6.20 (1H, d, J = 2.2 Hz, H-6).

#### Thioflavin-T (Th-T) binding assay

Th-T fluorescence assays were performed as described,^20^ using 10 μM Th-T. Crude extracts and all fractions were dried to remove native extraction solvents and reconstituted as 100× DMSO stocks (PGL-W also tested in water). Pure compounds (e.g., PGG, methyl gallate, luteolin) were prepared as 100× DMSO stocks. For all assays, stocks were diluted into the aqueous assay buffer to a fixed final vehicle of 1% (v/v) DMSO across all conditions, including controls. The control sample was defined as vehicle-matched, fibril-only (no test article). All wells received the same vehicle volume. Aged TTR and Aβ_1-42_ fibrils were incubated with compounds at 10, 50, or 100 μM for 24 h. Fluorescence (Ex 450 nm/Em 482 nm) was measured on an FP-8500 spectrofluorometer (JASCO, Tokyo, Japan). 1,4-naphthoquinone and KM001 served as positive and negative controls, respectively.^20^

#### Transmission electron microscopy (TEM)

Fibril morphology (±compound) was examined by TEM as described.^20^ Samples were applied to carbon-coated copper grids (STEM Cu100P; Okenshoji, Tokyo, Japan) for 5 min, negatively stained with 0.1% phosphotungstic acid for 3 min, air-dried 24 h, and imaged on a JEM-2100 (JEOL) at 80 kV.

#### SDS-PAGE and Coomassie brilliant blue staining

Tetramer and monomer fractions were analyzed by SDS-PAGE.^20^ Aged TTR samples were mixed with gel loading buffer (0.1% SDS, 13% glycerol). Non-boiled samples were resolved on 14% SDS-polyacrylamide gels and stained with Coomassie Brilliant Blue (Nacalai Tesque, Kyoto, Japan).

#### *C. elegans* health lifespan auto-monitoring system (C-HAS)

Automated monitoring of lifespan and healthspan was performed as described.^30^^,^^31^^,^^32^ Briefly, 2 mL NGM containing 2′-deoxy-5-fluorouridine (FUdR) was dispensed into 6-well plates. Synchronized L4 animals were transferred and imaged every 12 h using the Incucyte® S3 at 23°C. By overlapping consecutive images, changes in position and posture were detected. Animals showing no change in both position and posture across two consecutive 12-h intervals (≥24 h) were classified as dead; posture-only change as frail; and both position and posture changes as active. This rule provides an objective time-window-based criterion; results were spot-checked by manual verification on randomly sampled wells. A prior study has also established automated survival/behavior scoring.^49^

#### Intrapopulation analysis of lifespan and healthspan in *C. elegans*

Intrapopulation analysis was performed as described.^32^^,^^33^ Individuals were classified into four groups relative to the control group’s mean lifespan and mean healthspan rate (healthspan/lifespan × 100%). Groups were color-coded: orange (unhealthy: shorter lifespan & lower healthspan rate), red (short-lived with higher healthspan rate), blue (long-lived with lower healthspan rate), green (healthy: longer lifespan & higher healthspan rate). Pie charts indicate group proportions.

#### Visualization of TTR deposits in *C. elegans*

Synchronized L4-stage *C. elegans* expressing TTR_81-127_ (10–20 animals/experiment) were cultured on NGM ±10 μM PGG for 7 days. On days 3 and 10 of adulthood, nematodes were anesthetized with 40 mM sodium azide (NaN_3_), mounted on glass slides, and imaged using a BZ-X710 fluorescence microscope (Keyence, Osaka, Japan). TTR deposits were quantified using BZ-Analyzer software (Keyence).

#### Cell viability assays

SH-SY5Y human neuroblastoma cells (see key resources table) were seeded at 2.5 × 10^5^ cells/well (96-well plates; 100 μL). After 24 h, the medium was replaced with 100 μL of PGG or PGG-treated/disrupted V30M TTR fibrils in phenol-red-free medium containing 0.1% DMSO. V30M TTR fibrils were generated by aging TTR at pH 4.4 and then adjusting the samples to pH 8.0 prior to treatment and cell exposure. After 24 h, 1% Triton X-100 (10 μL; 15 min) served as negative-viability control. Cell Counting Kit-8 reagent (10 μL; Dojindo) was added for 3 h at 37°C. Absorbance at 450 nm was read on a BioTek microplate reader.^50^

#### Isolation of cardiac amyloid fibrils

As in our previous reports,^19^ cardiac amyloid fibrils were newly isolated from autopsied ATTR V30M tissue using the same buffer system and extraction sequence, under our current prospective protocol (approval no. 2651). Briefly, 150 mg of non-fixed frozen tissue was minced and washed with Tris-calcium buffer (20 mM Tris, 138 mM NaCl, 2 mM CaCl_2_, 0.1% NaN_3_, pH 8.0), then incubated overnight at 37°C with Clostridium histolyticum collagenase (5 mg/mL; Sigma). The pellet was resuspended in Tris-EDTA buffer (20 mM Tris, 140 mM NaCl, 10 mM EDTA, 0.1% NaN_3_, pH 8.0) and homogenized by repeated short bursts. After repeated centrifugations/supernatant removals, the final pellet was homogenized in ice-cold water and centrifuged; this water extraction was repeated 10 times, and supernatants were pooled as the water-soluble amyloid fractions. Samples were stored at 4°C until processing to minimize degradation; we note the single-donor origin and storage duration as limitations for generalizability.

### Quantification and statistical analysis

Unless stated otherwise, data are presented as mean ± SD. For group comparisons versus a single control, we performed one-way ANOVA with Dunnett’s multiple comparisons test and report *p* values together with 95% confidence intervals (CI) for mean differences.

In ThT assay, fluorescence was normalized to the vehicle control (set to 100%). Each condition was run in technical triplicate (*n* = 3 per experiment). PGG-treated experiments were independently repeated ≥2 times (biological replicates). To aid interpretation, standardized mean differences are summarized as Hedges’ g (bias-corrected Cohen’s d) with two-sided 95% CI (noncentral-t method; BCa bootstrap, 5,000 resamples, where noted). The effect-size CI was not multiplicity-adjusted. Effect sizes were computed using DABEST (estimation statistics).^48^ As a sensitivity check to variance heterogeneity, we also report Cohen’s d (95% CI). These results are summarized in Table S1 with the following fields: n, Mean (%), SD (%), Mean difference (percentage points, pp), *p* (Dunnett’s test), 95% CI (Dunnett’s test), Hedges’ g (95% CI), and Cohen’s d (95% CI).

All hypothesis tests were two-sided with significance set at *p* < 0.05. ANOVA/Dunnett procedure was carried out in GraphPad Prism 10 (GraphPad Software, San Diego, CA, USA), and *p*-value was shown in each figure legend; effect sizes were calculated with DABEST, and Hedges’ g (95% CI) was described in the results section.

## Supplemental information

Document S1. Figures S1–S5

Table S1. Descriptive statistics and treatment-control contrasts (ANOVA/Dunnett *p* and 95% CI), with standardized effect sizes (Hedges’ g, Cohen’s d)
