# Supplementary material for: Glycosidic scaffold bearing multiple galloyl moieties from pomegranate disrupts transthyretin amyloids
Source: iScience. 2025 Nov 21;29(1):114170. doi: 10.1016/j.isci.2025.114170 (PMC12765163; doi:10.1016/j.isci.2025.114170)
Supplement: Document S1. Figures S1–S5 [file mmc1.pdf]

iScience, Volume *29*

Supplemental information

Glycosidic scaffold bearing multiple galloyl moieties from pomegranate disrupts transthyretin amyloids

Asuka Kagami, Nami Hashimoto, Ryoko Sasaki, Yutaro Fukushima, Hari Prasad Devkota, Shoya Tanaka, Mikiyo Wada, Kunitoshi Yamanaka, Shiori Yamakawa, Shogo Misumi, Takeshi Yokoyama, Mineyuki Mizuguchi, Takashi Sato, Teruya Nakamura, Shunsuke Kotani, Mary Ann Suico, Hirofumi Kai, Mitsuharu Ueda, and Tsuyoshi Shuto

A

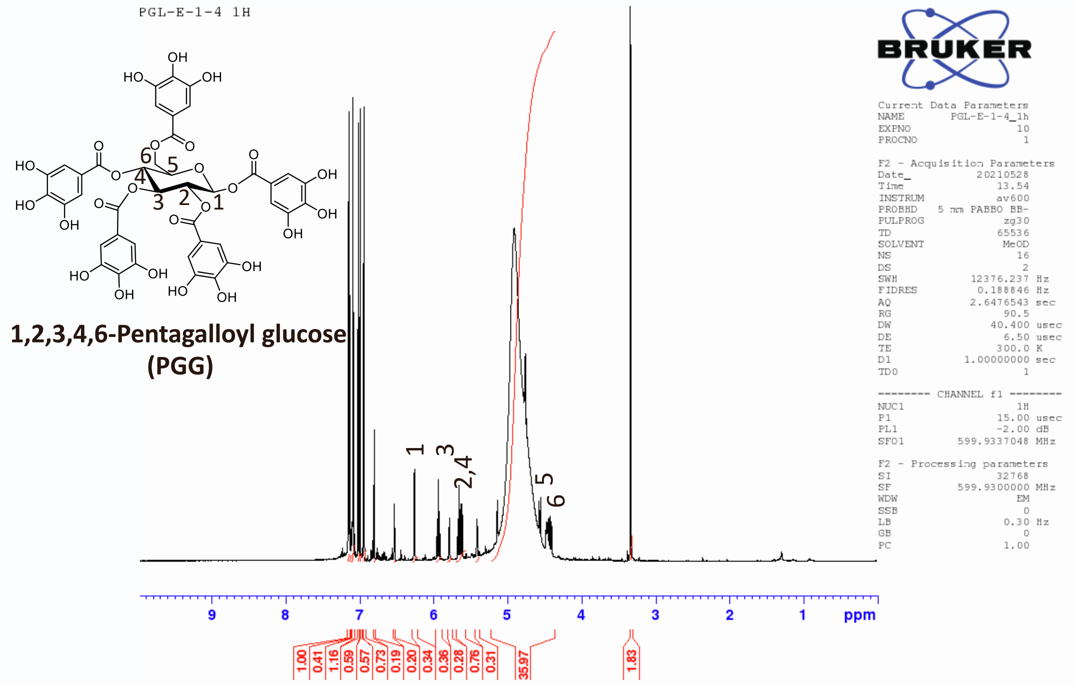

B

	Sample	Standard[23]
δ_H	7.12(2H, s, H-2’”/6’”)	7.14(2H, s, H-2’”/6’”)
	7.06(2H, s, H-2’/6’)	7.09(2H, s, H-2’/6’)
	6.99(2H, s, H-2””/6””)	7.01(2H, s, H-2””/6””)
	6.96(2H, s, H-2’””/6’””)	6.98(2H, s, H-2’””/6’””)
	6.91(2H, s, H-2”/6”)	6.94(2H, s, H-2”/6”)
	6.25(1H, d, J=8.0 Hz, H-1)	6.26(1H, d, J=8.0 Hz, H-1)
	5.91(1H, t, H-4)	5.90(1H, m, H-4)
	5.63(1H, m, H-5)	5.65(1H, m, H-5)
	5.59(1H, m, H-2)	5.61(1H, m, H-2)
	4.43(1H, m, H-3)	4.42(1H, m, H-3)
	4.39(2H, m, H-6)	4.39(2H, m, H-6)

C

	Sample	Standard[23]
δc	93.9(C-1)	93.8(C-1)
	74.1(C-3)	74.1(C-3)
	72.3(C-4)	72.2(C-4)
	69.8(C-2)	70.8(C-2)
	68.8(C-5)	68.4(C-5)
	63.1(C-6)	62.2(C-6)
	Galloyl i:	Galloyl i:
	119.8(C-1’)	119.7(C-1’)
	110.3(C-2’/6’)	110.3(C-2’/6’)
	140.0(C-4’)	140.0(C-4’)
	146.3(C-3’/5’)	146.2(C-3’/5’)
	166.3(C-7’)	166.2(C-7’)
	Galloyl ii:	Galloyl ii:
	120.2(C-1”)	120..2(C-1”)
	110.4(C-2”/6”)	110.3(C-2”/6”)
	140.1(C-4”)	140.1(C-4”)
	146.4(C-3”/5”)	146.4(C-3”/5”)
	167.0(C-7”)	166.9(C-7”)
	Galloyl iii:	Galloyl iii:
	121.1(C-1’”)	121.1(C-1’”)
	110.7(C-2’”/6’”)	110.7(C-2’”/6’”)
	140.8(C-4’”)	140.8(C-4’”)
	146.6(C-3’”/5’”)	146.5(C-3’”/5’”)
	168.0(C-7’”)	167.9(C-7’”)
	Galloyl iv:	Galloyl iv:
	120.3(C-1””)	120.2(C-1””)
	110.5(C-2””/6””)	110.4(C-2””/6””)
	140.4(C-4””)	140.3(C-4””)
	146.5(C-3””/5””)	146.4(C-3””/5””)
	167.0(C-7””)	167.0(C-7””)
	Galloyl v:	Galloyl v:
	120.4(C-1’””)	120.2(C-1’””)
	110.5(C-2’””/6’””)	110.4(C-2’””/6’””)
	140.4(C-4’””)	140.3(C-4’””)
	146.5(C-3’””/5’””)	146.4(C-3’””/5’””)
	167.3(C-7’””)	167.0(C-7’””)

Fig S1 Spectroscopic characterization of the isolated compound PGG
(A) ^{1}H NMR spectrum (CD_3OD).
(B) Comparison of ^{1}H NMR chemical shifts with literature values [23].
(C) Comparison of ^{13}C NMR chemical shifts with literature values [23].

[23] Taiwo, B.J., Popoola, T.D., van Heerden, F.R., and Fatokun, A.A. (2020). Pentagalloylglucose, isolated from the leaf extract of *Anacardium occidentale* L., could elicit rapid and selective cytotoxicity in cancer cells. BMC Complement. Med. Ther. 20, 287. https://doi.org/10.1186/s12906-020-03075-3.

A

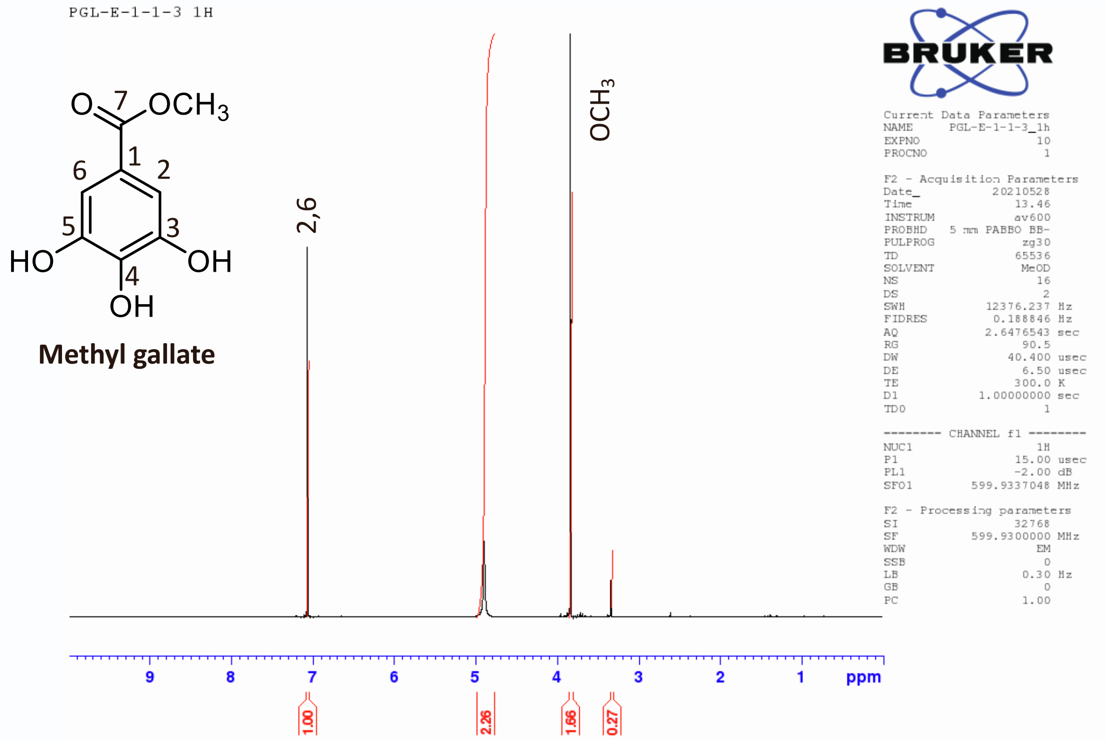

B

	Sample	Standard[22]
δ_H	7.06(2H, s, H-2, H-6)<br>3.83(3H, s, OCH_3)	7.05(2H, s, H-2, H-6)<br>3.81(3H, s, OCH_3)

C

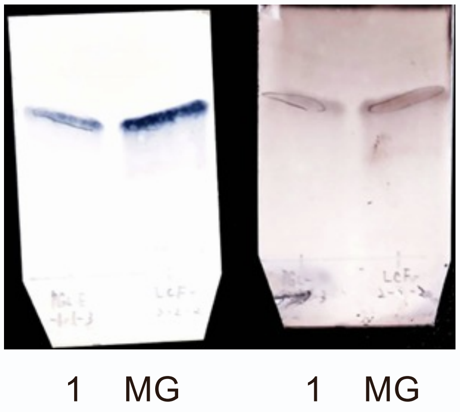

Fig S2 Spectroscopic characterization of the isolated compound Methyl gallate

(A) ^{1}H NMR spectrum (CD_3OD).

(B) Comparison of ^{1}H NMR chemical shifts with literature values [22].

(C) TLC (CHCl3:MeOH:Water=9 :1 :0.1,$FeCl_3$,H2SO4) 1: Sample, MG: Methyl gallate

[22] Lee, S.C., Kwon, Y.S., Son, K.H., Kim, H.P., and Heo, M.Y. (2005). Antioxidative constituents from *Paeonia lactiflora*. Arch. Pharm. Res. *28*, 775–783. https://doi.org/10.1007/BF02977342.

A

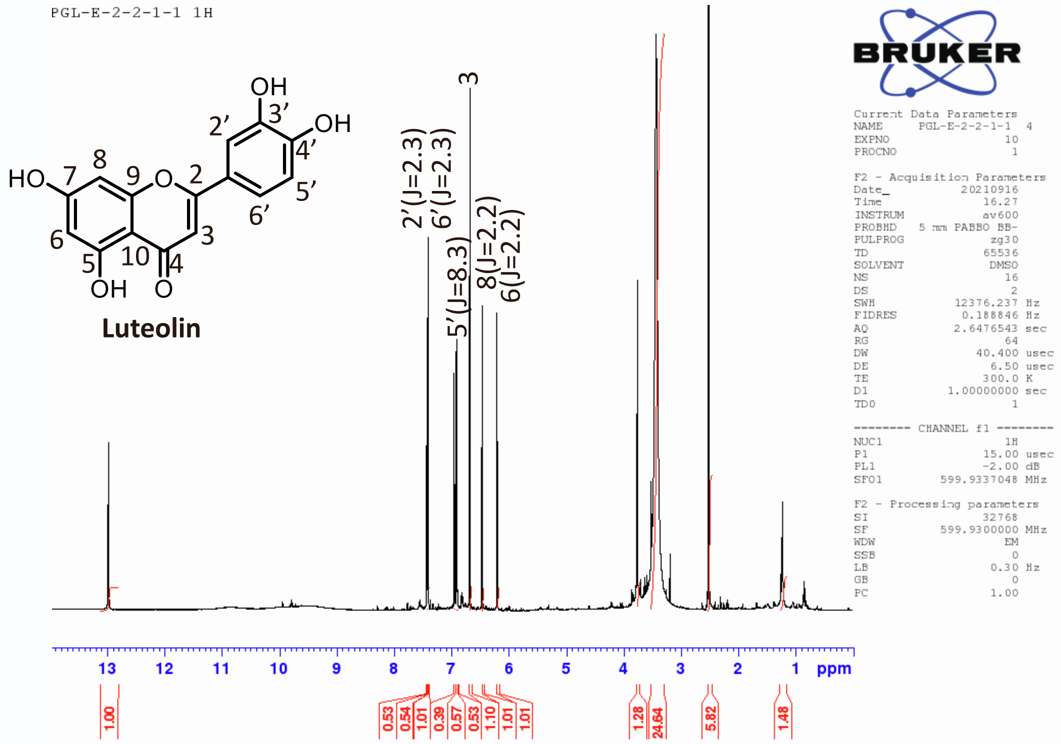

B

sd	Sample	Standard[24]
δ_H	7.41(2H, m, J = 2.3 Hz, H-2',H-6')<br>6.90(1H, d, J = 8.3 Hz, H-5')<br>6.68(1H, s, H-3)<br>6.46(1H, d, J = 2.2 Hz, H-8)<br>6.20(1H, d, J = 2.2 Hz, H-6)	7.36 (2H, m, H-2', H-6')<br>6.88 (1H, d, J = 8.5 Hz, H-5')<br>6.52 (1H, s, H-3)<br>6.42 (1H, d, J = 2.0 Hz, H-8)<br>6.19 (1H, d, J = 2.0 Hz, H-6)

Fig S3 Spectroscopic characterization of the isolated compound Luteolin
(A) ^{1}H NMR spectrum (CD_3OD).
(B) Comparison of ^{1}H NMR chemical shifts with literature values [24].

[24] Lin, L.-C., Pai, Y.-F., and Tsai, T.-H. (2015). Isolation of Luteolin and Luteolin-7-*O*-glucoside from *Dendranthema morifolium* Ramat Tzvel and Their Pharmacokinetics in Rats. J. Agric. Food Chem. 63, 7700–7706. https://doi.org/10.1021/jf505848z.

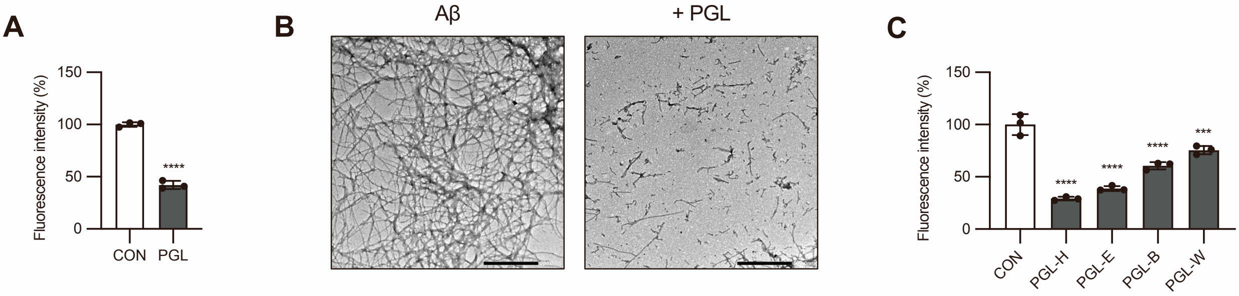

Fig S4 PGL disrupts $A\beta_{1-42}$ amyloid fibrils

(A) $A\beta_{1-42}$ amyloid fibrils were incubated with 0.5 mg/ml PGL for 24 h and analyzed using the Th-T fluorescence assay.

(B) TEM images corresponding to (A).

(C) $A\beta_{1-42}$ fibrils were treated with 0.5 mg/ml of PGL-H, PGL-E, PGL-B, or PGL-W fractions for 24 h and analyzed by Th-T assay.

Data shown are representative of three independent experiments and are presented as mean ± S.D. Statistical significance was determined using Student's *t*-test (A) (****$P <$ 0.0001 vs. control) and Dunnett's test (C) (***$P < 0.001$, ****$P < 0.0001$ vs. control).

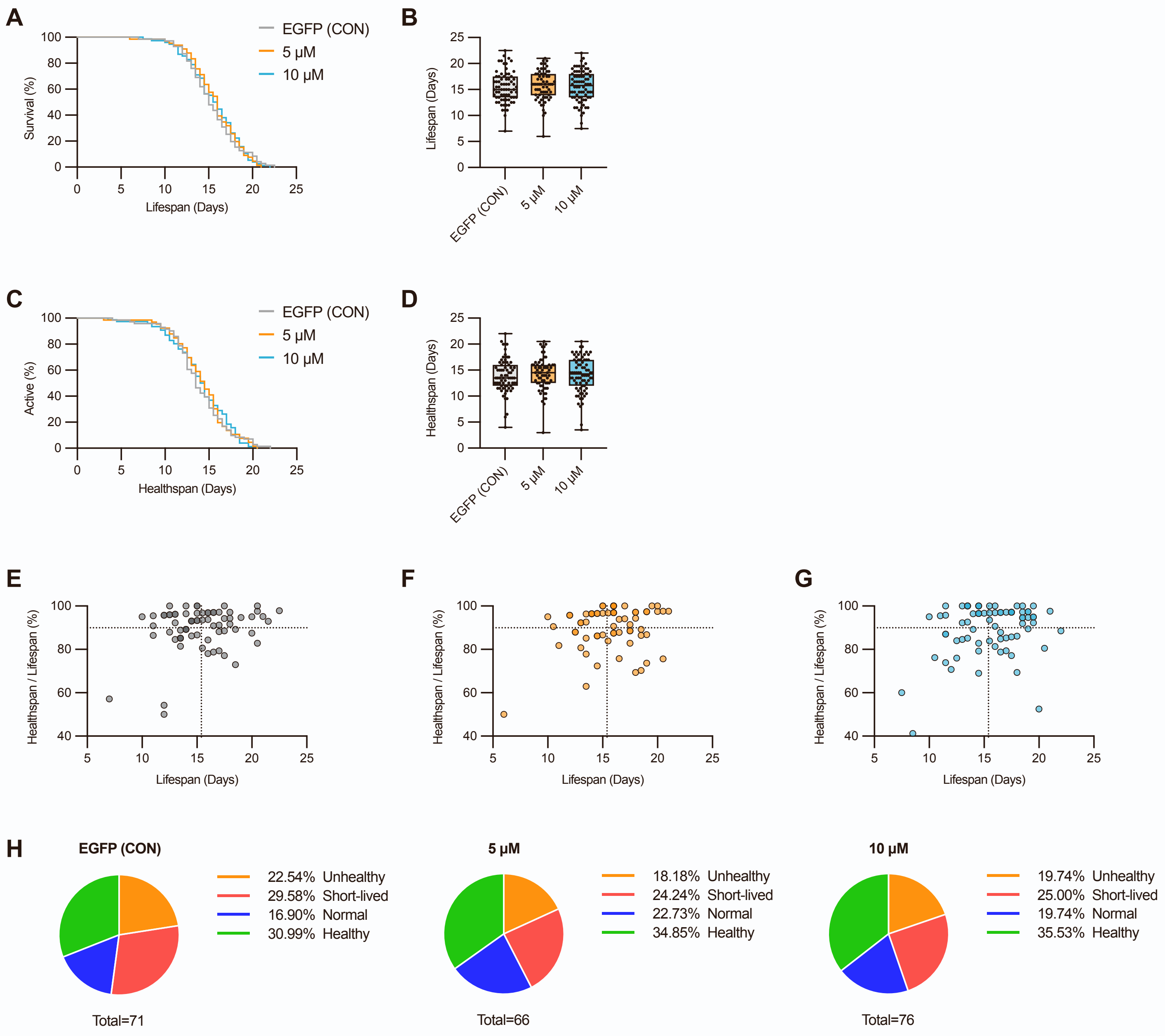

Fig S5 PGG does not alter lifespan or healthspan in EGFP expressing *C. elegans*

(A and C) Lifespan (A) and healthspan (C) curves of EGFP nematodes treated with 5 or 10 μM PGG (Control, n = 71; 5 μM, n = 66; 10 μM, n = 76).

(B and D) Box plots of lifespan (B) and healthspan (D) in EGFP nematodes treated with PGG, based on three independent biological replicates.

(E-G) Scatter plots showing individual lifespan and healthspan-to-lifespan ratio (%). Dashed lines on the X and Y axes represent the mean values of the control group.

(H) Pie charts showing the distribution of nematodes among predefined lifespan/healthspan categories.

Graphs are representative of three independent experiments. Data are presented as mean ± S.D.
